# Mesenchymal stem/stromal cells- a principal element for tumour microenvironment heterogeneity

**DOI:** 10.3389/fimmu.2023.1274379

**Published:** 2023-10-09

**Authors:** Li Sun, Yongliang Yao

**Affiliations:** Department of Clinical Laboratory, Kunshan First People’s Hospital, Affiliated to Jiangsu University, Kunshan, China

**Keywords:** tumor microenvironment, heterogeneity, cancer-associated mesenchymal stem/stromal cells, stemness, immune cells

## Abstract

The heterogeneity of the tumor microenvironment (TME) is a major obstacle in cancer treatment, making most therapeutic interventions palliative rather than curative. Previous studies have suggested that the reason for the low efficacy of immunotherapy and the relapse of the original responders over time may be due to the complex network of mesenchymal stem/stromal cells (MSCs), a population of multipotent progenitor cells existing in a variety of tissues. Cancer-associated MSCs (CA-MSCs) have already been isolated from various types of tumors and are characterized by their vigorous pro-tumorigenic functions. Although the roles of CA-MSCs from different sources vary widely, their origins are still poorly understood. Current evidence suggests that when local resident or distally recruited MSCs interact with tumor cells and other components in the TME, “naïve” MSCs undergo genetic and functional changes to form CA-MSCs. In this review, we mainly focus on the multiple roles of CA-MSCs derived from different sources, which may help in elucidating the formation and function of the entire TME, as well as discover innovative targets for anti-cancer therapies.

## Introduction

The International Society for Cellular Therapy issued a set of minimal standards for the recognition of mesenchymal stem/stromal cells (MSCs) in 2006 based on three principles: Firstly, in typical culture settings, MSCs must adhere to the plastic. Secondly, it is essential that MSCs express CD105, CD90 and CD73, while they must be negative for CD 45, CD 34, CD 14 or 11b, CD 79α or 19, and HLA-DR. Lastly, MSCs are capable of differentiating into adipocytes, chondrocytes, and osteoblasts under certain circumstances (1–3). In healthy tissues, MSCs play a key role in wound healing and immune regulation. In the bone marrow niche, MSCs play an important role in hematopoietic stem cell maintenance and lineage determination by secreting factors, and contribute to the construction of the niche (4). Although MSCs account for a relatively small proportion of the tumor microenvironment (TME), with studies reporting that they make up 0.01–5% of the total cell population, they can amplify their effects by directly altering the matrix composition of the TME and influencing the behavior of their offspring given the capacity for multi-directional differentiation (4–7).

It has been found that MSCs that reside within tumor tissue have high tumorigenicity compared to MSCs from healthy tissue (8). In-depth knowledge of the origin, heterogeneity, and functions of MSCs is critical to understanding disease progression and elucidating potential targets for novel therapeutic interventions.

## Origins of CA-MSCS

MSCs are a heterogeneous group of progenitor cells with both pro-tumor and anti-tumor effects (9–11). MSCs are recruited from adjacent tissues or circulating bone marrow to the tumor sites and reprogrammed into cancer-associated MSCs (CA-MSCs). Current evidence suggests that both recruited and local resident MSCs are major sources of CA-MSCs (12).

## The recruitment and reprogramming of MSCS

Chemokines and cytokines secreted by tumor cells and the surrounding stroma have been shown to be involved in the mechanisms of migration or homing of the circulating bone marrow MSCs (BMMSCs) to the TME. It is well known that tumor-produced CCL2, CCL5, CXCL12 and CXCL16 play crucial roles in the chemotaxis of BMMSCs (13). In addition, cytokines such as PDGF, TGF-β, VEGF and TNF-α were also found to play important roles in BMMSCs homing (14, 15). For example, a study in prostate cancer showed that TGF-β1 is a key molecule that regulates the distant recruitment of BMMSCs into tumor sites (16). MSCs present in adjacent normal tissues express the homing spectrum of inflammatory signals from tumor cells, and are thus anchored in the tumor mass (17). However, the signaling pathways and underlying mechanisms by which MSCs migrate from adjacent normal tissues to tumor sites are still unknown.

Based on evidence, tumor cells can effectively reprogram the phenotype and function of “naïve” MSCs. Le et al. have demonstrated that ovarian cancer cells cause “naïve” MSCs to express CD73, CD90 and CD105, while CD14, CD20, CD34 and CD45 are absent. Moreover, the levels of CXCL1, CXCL2 and IL-8 secreted by MSCs increase through PI3K/Akt, MAPK and NF-κB signaling pathways, which in turn make tumor cells resistant to chemotherapy and the differentiation of monocytes into M2 type (18). Wang et al. have shown that gastric cancer cells induce “naïve” mesenchymal stem cells have pro-metastatic behavior by activating YAP signaling through the exosomal Wnt5a (19). Exosomes derived from gastric cancer cell cause abnormal activation of the NF-kB signaling pathway in MSCs, thus promoting the secretion of pro-inflammatory factors by macrophages and the activation of CD69 and CD25 on the surface of T cells (20). In addition to the tumor cells, the surrounding microenvironment also plays a critical role in the malignant transformation of “naïve” MSCs into CA-MSCs. For example, mononuclear/macrophage-derived TNF-a makes “naïve” MSCs similar to CA-MSCs in their chemokine profile and ability to promote tumorigenicity in lymphomas (21). Moreover, neutrophils treated with tumor cells activate the AKT and p38 pathways in “naïve” MSCs by secreting inflammatory factors such as IL-17, IL-23 and TNF-α, and transform them into CA-MSCs, thus significantly promoting the growth and metastasis of gastric cancer (22).

## MSCS naturally residing in the TME

**Figure 1:**
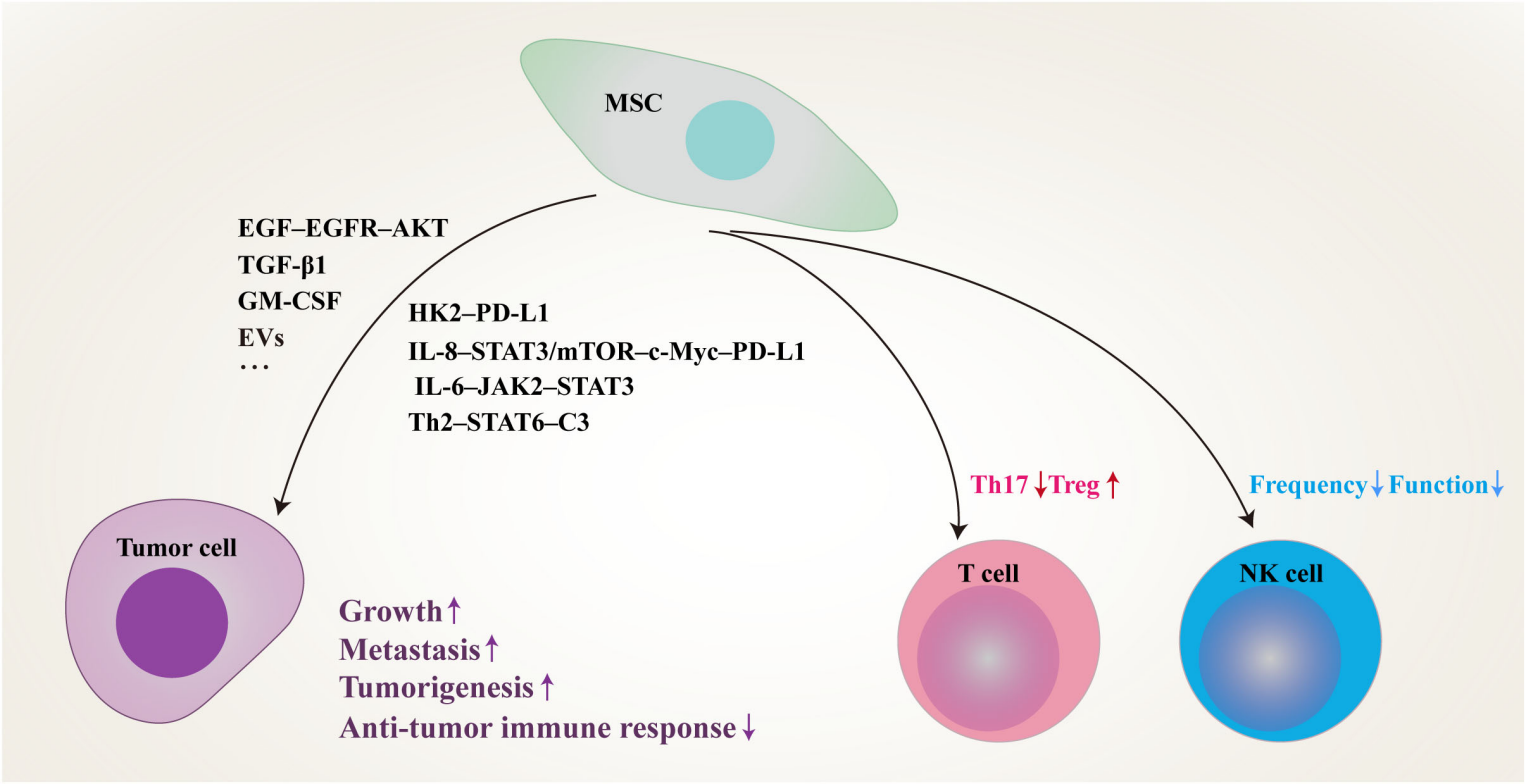
Schematic of proposed network of MSCs residing in TME inhibit anti-tumor immune responses and promote tumor progression by modulating tumor cells and immune cells through multiple signaling pathways. MSC, mesenchymal stem/stromal cell; PD-L1, programmed death-1 ligand; EVs, extracellular vesicles; IL, interleukin; STAT, signal transducer and activator of transcription; EGF, epidermal growth factor; EGFR, epidermal growth factor receptor; TGF-β, transforming growth factor-β; GM-CSF, granulocyte-macrophage colony-stimulating factor; HK2, hexokinase 2; mTOR, mammalian target of rapamycin; JAK2, janus kinase 2; Th, T helper; C3, complement 3; Treg, regulatory T cell; NK, natural killer.

As one of the key stromal cell types in the tumor niche, CA-MSCs have been isolated from various tumors and have been found to play an important role in tumor progression (**Table 1**). MSCs isolated from gastric cancer tissues (GCMSCs) promote cancer progression and contribute to tumor immunotherapy tolerance through the CXCR2–HK2–PD-L1 pathway (23). IL-8 derived from GCMSCs induces the expression of PD-L1 in GC cells through c-Myc regulated by the STAT3 and mTOR signaling pathways and inhibits the anti-tumor immune response (24). GCMSCs also disrupt the Treg/Th17 balance in peripheral blood mononuclear cells (PBMCs) by inhibiting Th17 cell proliferation and inducing Tregs differentiation, thereby impairing the anti-tumor immune response (25). GCMSCs reduce the frequency of infiltrating NK cells and inhibit their effector function to promote tumor growth through the mTOR signaling pathway (26). Lung cancer-associated MSCs exert strong metastasis-promoting effects by producing complement C3, which is induced and maintained by Th2 cytokines in a STAT6-dependent manner and also promote tumor metastasis and tumorigenesis by induction of EMT and stem-like reprogram (27, 28). Single-cell transcriptional analysis of MSCs in invasive breast cancer TME revealed increased expression of the COL10A1 and COL8A1 genes, as well as pro-tumor effects driven by TGF-β-related signals (29). Breast cancer-associated MSCs also promote cancer proliferation and enhance mammosphere formation partially via EGF–EGFR–AKT pathway (30). MSCs residing in other solid tumors also reported to contribute to tumor progression, such as pancreatic cancer, cervical cancer, colon cancer, head and neck squamous cell cancer and neuroblastoma (31–35). In general, MSCs residing in TME not only up-regulate the proliferation, migration and invasion ability of tumor cells and their PD-L1 expression, but also disrupt the balance between immune cells, thus inhibiting the anti-tumor immune response (**Figure 1**).

**Table 1:** The functions and signaling pathways of MSCs naturally residing within the TME.

Tumour type	Signalling pathway	Function	Reference
Gastric cancer	CXCR2–HK2–PD-L1 IL-8–STAT3/mTOR–c-Myc N/A mTOR	Induce lactate overproduction in cancer cells and impaired function of CD8^+^ T cells Promote cancer cells express PD-L1 Inhibit Th17 cell proliferation and induce Tregs differentiation Inhibit the frequency and effector function of infiltrating NK	REF. (23) REF. (24) REF. (25) REF. (26)
Lung cancer	Th2–STAT6–C3 N/A	Promote neutrophil recruitment and facilitate cancer cells migration Induce EMT and stemness of cancer cells	REF (27) REF (28)
Breast cancer	TGF-β EGF–EGFR–AKT	Inhibit the proliferation of cancer cells Promote mammosphere formation of cancer cells	REF. (29) REF. (30)
Pancreatic cancer	GM-CSF	Enhance the proliferation, invasion and migration potential of cancer cells	REF. (31)
Cervical cancer	TGF-β1	Induce 5’-nucleotidase expression and produce immunosuppressive nucleoside of cancer cells	REF. (32)
Colon cancer	IL-6–JAK2–STAT3	Promote the proliferation and migration of cancer cells	REF. (33)
Head neck squamous cell cancer Neuroblastoma	Indoelamine 2,3 dioxygenase Indoelamine 2,3 dioxygenase and PGE2	Abrogate proliferation and cytokine production of CD4^+^ and CD8^+^ T cells Inhibit the cytolytic activity of NK Cells	REF. (34) REF. (35)

N/A, not available.

Besides solid tumors, hematological malignancies also rely heavily on support of host cells, specifically MSCs (36). The development of multiple myeloma MSCs (MM-MSCs) may be the result of multiple factors, and changes may vary depending on the individual, lesion location, co-culture myeloma cell type, and cell subsets within the MSC population. MM-MSCs differ from non-diseased donors (ND-MSCs) in a number of ways, including cytokine production. MM-MSCs secrete higher levels of growth factors such as SCF, VEGF, and IL-6, than ND-MSCs. Recent studies have shown that, compared with ND-MSCs, MM-MSCs respond to myeloma cells with increased expression of IL-10, the TNF family member B-cell activating factor, and HGF (37, 38). MM-MSCs, but not ND-MSCs, can also induce bortezomib resistance via enhanced NF-κB activity in MM cells (39). MM-MSCs are spatially co-located with tumor cells and immune cells which express genes involved in tumor survival and immune regulation. Inflammatory MSCs (iMSCs) are specific for MM bone marrow. The development of iMSCs is the result of NF-κB signaling activated by inflammatory mediators (40).

## Different properties between CA-MSCS and “naïve” MSCS

Interactions with tumor cells and other components in the TME lead to genetic and functional changes in “naïve” MSCs (18, 41). CA-MSCs show stronger proliferation and migration abilities and have stronger tumor-promoting effects than “naïve” MSCs. In addition, the number of G0/G1 phase cells is increased in CA-MSCs compared to their counterparts in neuroblastoma, suggesting that CA-MSCs play an important role in regulating cancer dormancy. Compared with “naïve” MSCs, CA-MSCs also exhibit different adipogenic differentiation and immunomodulatory abilities (12).

## Regulatory effects of MSCS from different sources on tumor cells

Evidence indicates that the specific effects of MSCs on tumors depend on the origin and tumor type (**Table 2**).

**Table 2:** The phenotypes and functions of MSCs on tumor cells from different sources.

MSC source	Phenotype	Tumour type	Function	Reference
Bone marrow	Positive for CD13, CD44, CD73, CD90, CD105, CD166, STRO-1 and negative for CD14, CD34, CD45	Lung cancer Pancreatic cancer Prostate cancer Breast cancer Osteosarcoma Colon cancer	Promote metastasis Enhance the clonogenic potential of cancer cells Short-term pro-apoptosis, long-term promote chemo-resistance Enhance the stemness of cancer cells Enhance the stemness and migration capacity of cancer cells Increase the population of CSCs	REF. (3, 42) REF. (43) REF. (44) REF. (45, 46) REF. (47) REF. (48)
Umbilical cord	Positive for CD13, CD29, CD63, CD73, CD90, CD105, HLA-ABC and negative for CD31, CD34, CD45, CD133, CD271, HLA-DR	Lung cancer Glioblastoma Hepatocellular cancer Colorectal cancer	Promote the proliferation and migration of cancer cells, resist apoptosis and autophagy Inhibit the proliferation, migration and invasion of cancer cells and promote their apoptosis Reduce the proliferation and apoptosis of cancer cells and inhibit angiogenesis Inhibit the proliferation, migration and invasion of cancer cells	REF. (2, 49–51) REF. (52) REF. (53) REF. (54, 55)
Adipose tissue	Positive for CD9, CD13, CD29, CD44, CD54, CD73, CD90, CD105, CD106, CD146, CD166, HLA I, STRO-1 and negative for CD11b, CD14, CD19, CD31, CD34, CD45, CD79α, CD133, CD144, HLA-DR	Melanoma Hepatocellular cancer Colon cancer Head and neck cancer Breast cancer Glioblastoma	Promote the proliferation, migration and invasion of cancer cells Increase the efficacy of chemotherapy Promote the proliferation, migration and invasion of cancer cells Promote the proliferation, and migration of cancer cells Inhibit the proliferation and migration, enhance apoptosis and induce PD-L1 expression of cancer cells Inhibit the proliferation, invasion and angiogenesis of cancer cells	REF. (3, 56) REF. (57) REF. (58) REF. (59) REF. (60, 61) REF. (62)

### Bone marrow MSCs

BMMSCs promote metastasis by activating the ABL–MMP9 signaling axis in lung cancer cells (42). BMMSCs secreted IL-6 contribute significantly to pancreatic cancer growth (43). However, BMMSCs-derived IL-28 trigger prostate cancer cell apoptosis via IL-28Rα–STAT1 signaling pathway (44). The plasticity of cancer stem-like cells (CSCs) is one of the primary elements responsible for the heterogeneity of the TME (63). Estrogen receptor-positive breast cancer cells make direct contact with BMMSCs and acquire CSCs phenotype with increased resistance to standard antiestrogenic drugs (45). Extracellular vesicles released by BMMSCs instruct breast cancer cells to become dormant CSCs with chemoresistance capacity (46). Cancer cell-stimulated BMMSCs also create a CSCs niche via the release of prostaglandin E2 (64). IL-6 secreted by tumor-stimulated BMMSCs, enhances the stemness and migration capacity of osteosarcoma cells (47). BMMSCs-derived exosomes promote colon CSCs traits via miR-142-3p (48).

### Umbilical cord MSCs

Umbilical cord MSCs (UCMSCs) are known to facilitate the proliferation and movement of lung cancer cells through the ERK–phospho-c-Fos-S374 pathway and to induce malignant characteristics of cancer cells through the AMPK signaling pathway (49, 50). UCMSCs inhibit the growth and metastasis and promote the apoptosis of lung cancer cells by regulating the PI3K–Akt and NF-κB pathways (51). UCMSCs inhibit the proliferation, migration and invasion of glioblastoma cells by regulating the IL-6–JAK2–STAT3 signaling pathway, and promote the apoptosis of glioblastoma cells (52). Exosomes derived from UCMSCs have anti-proliferative, pro-apoptotic, and anti-angiogenic effects on hepatocellular carcinoma cells (53). Exosomes secreted by UCMSCs also inhibit macrophage M2 polarization and prevent liver metastasis of colorectal cancer by inducing miR-1827 to target SUCNR1 (54). Regulation of ITGA2/ITGA6 has been demonstrated to be the means by which UCMSCs suppress the proliferation, migration and invasion of colon cancer cells (55).

### Adipose tissue-derived MSCs

Adipose tissue-derived MSCs (ADMSCs) can be used in the treatment of lung metastatic melanoma therapy by producing the anti-angiogenic factor TSP-1 (56). ADMSCs can increase the efficacy of chemotherapy in hepatocellular carcinoma treatment (57). ADMSCs also accelerate the progression of colon cancer by inducing an MSC-transformed cancer-associated fibroblast phenotype through the ICAM1–STAT3–AKT axis (58). ADMSCs promote the proliferation and migration of head and neck cancer cells (59). In addition, preclinical treatment of breast cancer is aided by ADMSCs, which exhibit anti-tumor activity (60). It has been demonstrated that CCL5 secreted by ADMSCs can induce PD-L1 expression in breast cancer cells (61). Exosomes derived from ADMSCs reduce glioblastoma proliferation and significantly down-regulate the invasion-related genes ITGα5 and ITGβ3, as well as the angiogenesis induction gene KDR (62).

### Naturally residing MSCs

IL-6 derived from ameloblastoma MSCs (AMMSCs) induces epithelial–mesenchymal transition (EMT) and promotes the formation of CSCs through the STAT3 and ERK1/2 signaling pathways (65). Endometrial cancer-derived MSCs exhibit high expression of PD-L1 and PD-L2, which can be augmented by TNF-α and IFN-γ (66). Additionally, our previous studies have revealed that IL-8 derived from GCMSCs could promote immune escape by inducing PD-L1 expression in GC cells via c-Myc, which is regulated by the STAT3 and mTOR signaling pathways (24). Moreover, our findings have shown that GCMSCs upregulate PD-L1 expression not only in the cell membrane of GC cells, but also in the cytoplasm and nucleus. Checkpoint blocking antibodies only block immune checkpoints on cell membranes. Thus, their effects may be counteracted by checkpoints from cytoplasmic translocation (67). The accumulation of nuclear PD-L1, which is promoted by deacetylation, has been reported to activate genes involved in the immune response (68). Evidence has suggested that PD-L1 expression is linked to tumor cells’ intrinsic properties (69). GCMSCs can also induce changes in PD-L1 expression by regulating the intrinsic metabolism of tumor cells (23). We have shown that GCMSCs can augment the CSCs properties of GC cells through PD-L1, thus leading to GC cells’ resistance to chemotherapy (67).

## Regulatory effects of MSCS from different sources on immune cells

**Figure 2:**
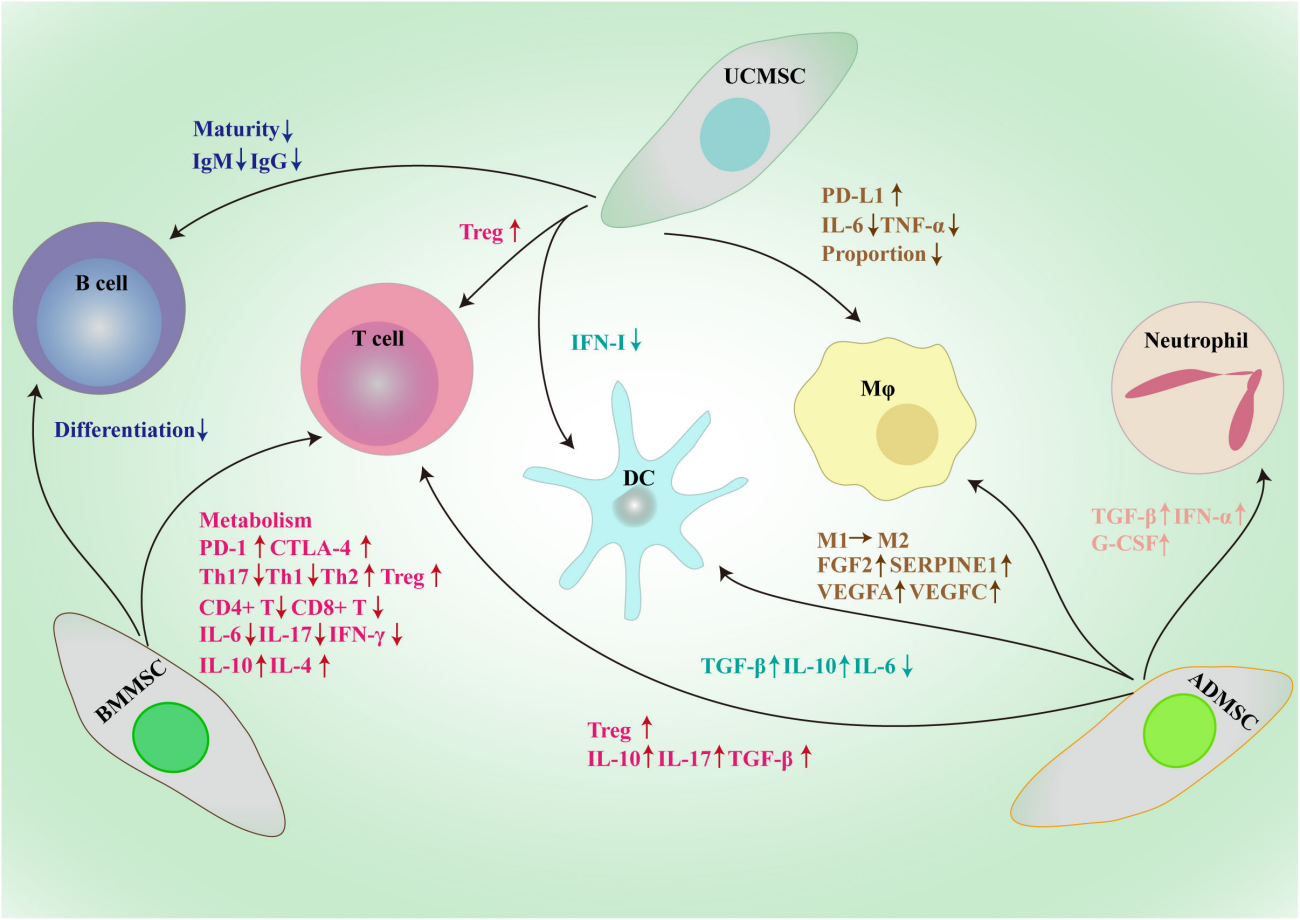
Framework diagram of MSCs derived from different sources exerting broad immunosuppressive effects through the interactions with immune cells, such as macrophages, dendritic cells, T cells, B cells and neutrophil. BMMSC, bone marrow mesenchymal stem/stromal cell; UCMSC, umbilical cord mesenchymal stem/stromal cell; ADMSC, adipose tissue-derived mesenchymal stem/stromal cell; PD-L1, programmed death-1 ligand; MΦ, macrophage; DC, dendritic cell; CTLA-1, cytotoxic T-lymphocyte antigen 4; Treg, regulatory T cell; Th, T helper; IL, interleukin; TGF-β, transforming growth factor-β; IFN, interferon; Ig, immunoglobulin; G-CSF, granulocyte colony-stimulating factor; FGF2, fibroblast growth factor 2; SERPINE1, serpin family E member 1; VEGF, vascular endothelial growth factor.

MSCs not only regulate tumor cells to boost the growth and progression, but also have effects on a variety of immune cells to modify the complex immune microenvironment (**Figure 2**).

### Bone marrow MSCs

The quantity and quality of tumor-killing T cells in the TME are essential for the success of immune therapeutics; however, due to the expression of immune checkpoints, the majority of infiltrating T cells are unable to identify and eliminate neighboring tumor cells (70, 71). The PD-1–PD-L1 axis is involved in the BMMSCs-mediated inhibition of T cell glycolysis by negatively regulating HK2 activity (72). IL-17 can also significantly increase the level of PD-L1 produced by BMMSCs through inducing iNOS expression to shape the immunosuppressive TME (73). Moreover, BMMSCs raise PD-1 expression on activated CD4^+^CD25^−^ T cells and induce apoptosis of these cells (74). Endothelial cells were safeguarded from cytotoxic T lymphocyte-induced lysis by co-culturing with BMMSCs (75). Besides, BMMSCs help create an immunosuppressive microenvironment in tumors by reducing the proportion of Th17 cells and increasing the proportion of Tregs in PBMCs (76). Studies have demonstrated that BMMSCs can drastically impede the growth of CD4^+^ and CD8^+^ T cells through cell-contact and paracrine-dependent means (77). Moreover, MSCs not only have an effect on T cell viability and proliferation, but also impede the production and operation of Th1 and Th17 cells, while encouraging the production of Th2 cells and Tregs (78, 79). Dendritic cells educated by BMMSCs initiate a transition from Th1 to Th2, stimulating Tregs and leading to a decrease in pro-inflammatory cytokines such as IL-6, IL-17, and IFN-γ, as well as an upsurge in IL-4 and IL-10 production (80). Another important soluble factor derived from BMMSCs is IL-1RA; BMMSCs from IL-1RA-deficient mice do not inhibit B cell differentiation (81).

### Umbilical cord MSCs

UCMSCs attenuate acute lung injury via PGE2-dependent reprogramming of macrophages to promote their PD-L1 expression (82). Besides, UCMSCs shift the cytokine profile of dendritic cells from proinflammatory to immunoregulatory (83). A study has reported that human UCMSCs disrupt the maturation of B cells by secreting TGF-β (84). Che et al. also have reported that UCMSCs inhibit the production of IgG and IgM in B cells (85). TGF-β secreted by UCMSCs enhances the induction of Tregs from “naïve” T cells to suppress colitis-associated colorectal cancer (86). UCMSCs inhibit the tumorigenesis of colon cancer by reducing the proportion of macrophages and inhibiting TNF-α and IL-6 secretion (87).

### Adipose tissue-derived MSCs

ADMSCs promote the activity of neutrophils by inhibiting apoptosis mediated by the increased expression of TGF-β, IFN-α, and G-CSF (88). Moreover, ADMSCs-derived exosomes enhance the secretion of TGF-β and IL-10 and impede the secretion of IL-6 by dendritic cells (89). ADMSCs upregulate FGF2, SERPINE1, VEGFA and VEGFC in macrophages and supporting the proliferation of breast cancer cells (90). Exosomes secreted by ADMSCs enable macrophages to switch from the M1 to the M2 phenotype, thus possessing potent anti-inflammatory properties (91). In the presence of ADMSCs, the proportion of Tregs increases and the production of IL-10, IL-17, and TGF-β by T cells is enhanced (92).

### Naturally residing MSCs

Endometrial cancer-derived MSCs inhibit the proliferation of PBMCs, which can be partially rescued by treatment with anti-PD-L1 antibodies (66). Glioma-associated MSCs enhance the immunosuppressive activity of MDSCs through a positive feedback loop of miR-21–SP1–DNMT1 (93). GCMSCs efficiently promote macrophage polarization toward the pro-tumor M2 subtype through several soluble molecules, including IL-6, IL-8, TGF-β, HGF, PGE2, IL-1RA, TSG6 and IDO (94). MSCs from head and neck squamous cell cancer inhibit the proliferation of CD4^+^ and CD8^+^ T cells through indoleamine 2,3 dioxygenase activity (34). GCMSCs inhibit the degranulation ability, perforin production, and cytotoxicity of NK cells by up-regulating the expression of fructose-bisphosphatase 1 (95). Moreover, Lung cancer-associated MSCs reduce NK cells cytotoxicity by expressing IL-6 and prostaglandin E2 (96).

## Conclusions

MSCs from different sources play various biological roles, and different tumors react in different ways to MSCs. MSCs not only manipulate the stemness of tumor cells, but also change the expression of immune checkpoint molecules on tumor cells and have huge effects on immune cells. Compared with “naïve” MSCs, CA-MSCs exhibit several unique characteristics, including stronger pro-tumor effects; they are considered to be key regulators of tumor fate. Given the multifaceted role that MSCs play in the TME, they represent an untapped but promising target for improving cancer therapies. It is necessary to formulate targeted therapy plans according to individual conditions in the treatment of cancer patients.

## Author contributions

LS: Funding acquisition, Writing – original draft. YY: Writing – review & editing.
